# Behind the developing brains and beating hearts of stem cell-derived embryo models

**DOI:** 10.1098/rsob.220325

**Published:** 2023-01-11

**Authors:** Gianluca Amadei, David M. Glover

**Affiliations:** ^1^ Department of Biology, University of Padua, Padua, Italy; ^2^ Department of Genetics, University of Cambridge, Cambridge, UK; ^3^ Division of Biology and Biological Engineering, California Institute of Technology, Pasadena, CA 91125, USA

**Keywords:** stem cells, embryo models, haematopoiesis

## Abstract

Studies over the past decade have shown how stem cells representing embryonic and extra-embryonic tissues of the mouse can self-assemble in the culture dish to recapitulate an astonishing part of early embryonic development. A systematic analysis has demonstrated how pluripotent embryonic stem cells can be induced to behave like the implanting epiblast; how they can interact with trophectoderm stem cells to form a patterned structure resembling the implanting embryo prior to gastrulation; and how the third stem cell type—extra-embryonic endoderm cells—can be incorporated to generate structures that undergo the cell movements and gene expression patterns of gastrulation. Moreover, such stem cell-derived embryo models can proceed to neurulation and establish progenitors for all parts of the brain and neural tube, somites, beating heart structures and gut tube. They develop within extra-embryonic yolk sacs that initiate haematopoiesis. Here we trace this journey of discovery.

## Graphical abstract

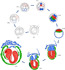

## 1. Embryonic and extraembryonic stem cells

A visitor to the Genetics Department in Cambridge in the late 1970s and early 1980s would be hit by the pervading smell of Martin Evans's mouse colony in the basement. At that time, Martin and Matthew Kaufman were in the process of establishing the first embryonic stem cell (ESC) lines, an achievement contemporaneous with similar work by Gail Martin at UCSF [1,2]. ESCs are pluripotent; they can generate any tissue of the body and so when re-introduced into embryos, they contribute to the formation of chimeric animals. Martin Evans was able to show that genetically modified ESCs could be introduced into mice in this way and the chimeric gonads could produce gametes able to transmit the transgene to future generations [3,4]. It was a short step to using this approach to knockout genes in ESCs by homologous recombination [5,6] and using these cells to generate transgenic knockout mice leading to the award of the Nobel Prize in 2007 to Martin Evans, Mario R. Capecchi and Oliver Smithies.

This long-standing knowledge that ESCs are pluripotent and so able to contribute to all tissues of a mouse when introduced back into their natural environment, the embryo, became the mainstay of the use of the mouse as a model system in which to study development or in which to replicate aspects of human disease. Others asked whether ESCs were not only capable of contributing to all tissues of the naturally developing mouse embryo, but also able to develop into three-dimensional structures that could replicate developmental events solely in culture. Broadly speaking these attempts have taken two paths. The first stemmed from observations in Roel Nusse's group that Wnt signalling could break symmetry and mediate aspects of formation of the embryonic anterior-posterior axis in ESC-derived embryoid bodies [7]. This system has been taken further, primarily by Alfonso Martinez-Arias's group, with the objective of trying to recapitulate some aspects of development starting solely from ESCs. By treating embryoid bodies with various exogenous compounds including activin A and a Wnt/β-catenin signalling agonist, the Martinez-Arias group were able to get the structures to initiate a programme of developmental events to form their so-called *gastruloids*. These structures could break symmetry, specify the germ layers and develop axial organization including the appropriate expression of Hox gene clusters notably in the absence of any extra-embryonic tissues [8–11]. Moreover, when embedded in Matrigel, gastruloids can be induced to form trunk-like structures with somites, a neural tube and a gut [12,13]. While remarkable, and giving new insight into the power of embryonic cells to organize into several well-defined structures, gastruloids do have limitations. Gastruloids do not faithfully replicate several morphological processes of embryogenesis, including the gastrulation movements *per se*, nor do they recapitulate development of the complete anatomy of natural embryos, particularly that of the brain.

In this article, our focus will be upon the second main approach to model embryo development that uses not just ESCs but also stem cells for the extra-embryonic tissues, which represent the two different extra-embryonic stem cell types present at the time the embryo implants into the uterus. This approach is based on a series of studies from Magdalena Zernicka-Goetz's lab initiated in 2013 and culminating in the use of cultured stem cells to generate embryoids that are able to undertake gastrulation and develop through neurulation to form progenitors of all brain regions, a neural tube, somites, a beating heart, gut tube progenitors and primordial germ cells all within a yolk sac that initiates haematopoiesis.

## 2. The three implantation lineages

**Figure 1.**
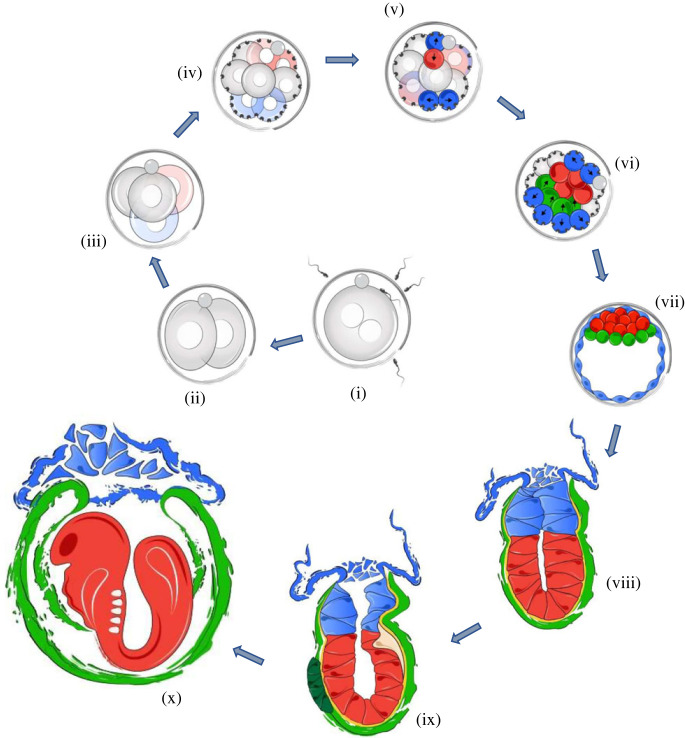
Development of the early mouse embryo. (i) Fertilized egg with male and female pronuclei. (ii) 2-cell embryo. (iii) 4-cell embryo in which cells begin to show bias towards their future fates. (iv) 8-cell embryo when cells become polarized. (v) 16-cell embryo in which a set of apolar cells have become internalized and retain pluripotency (red cells) whereas outer cells retain polarity and are destined to become trophectoderm (blue). (vi) Morula in which inner cell mass now comprises pluripotent epiblast cells (red) and primitive endoderm cells (blue). (vii) Blastocyst with expanded cavity and in which epiblast and primitive endoderm cells have sorted into distinct layers. (viii) Egg cylinder stage. The blastocyst has implanted into the uterus; the epiblast formed a rosette that has cavitated and elongated (red); the polar trophectoderm (overlaying the epiblast in the blastocyst) has become the extra-embryonic ectoderm (blue); the primitive endoderm has become visceral endoderm (green) which has spread to cover the structure. (ix) Gastrulation. Cells destined to become mesoderm (beige) move out of the epiblast layer to form the primitive streak which extends anteriorly. (x) Embryo (red) at E8.5 within the extra-embryonic endoderm (VE)-derived yolk sac (green) attached to placental tissue (blue). Drawings by Anna Hupalowska.

By the time the mouse blastocyst is ready to implant into the uterus, it comprises three cell types: pluripotent epiblast and two types of extra-embryonic tissue; primitive endoderm (PE), that will become visceral endoderm (VE) that gives rise to the yolk sac; and trophectoderm (TE), progenitor of the placenta (figure 1). ESCs represent the pluripotent epiblast that forms a compartment distinct from the extra-embryonic cells in the blastocyst. The epiblast arises from a set of cells internalized during the 8- to 16-cell and 16- to 32-cell cleavage stages, the *inner cell mass.* The cells remaining on the outside of the embryo at this time become the TE. Before implantation, a salt and pepper mixture of inside cells, now specified as PE and epiblast, sort from each other into distinct layers. Just as the epiblast is represented by ESCs, cultured stem cell lines of trophoblast stem cells (TSCs) have been established from the TE [14] and of eXtraEmbryoNic stem cells (XEN cells) from the PE/VE [15]. The trick in recapitulating development beyond gastrulation to form structures representing the entire body plan has been to recreate interactions between the pluripotent ESCs and the two extra-embryonic stem cell types.

## 3. The epiblast rosette—forming the amniotic cavity

**Figure 2.**
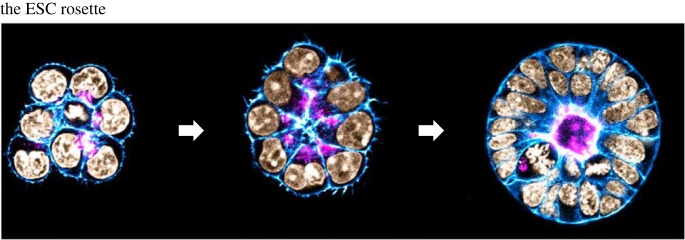
ESCs mimicking formation of the epiblast rosette. Aggregates of small numbers of ESCs plated into Matrigel transform into rosette-like arrangements that undergo cavitation, mimicking the formation of similar structures by the epiblast of the natural embryo upon implantation. Images by Marta Shahbazi.

Bedzhov & Zernicka-Goetz began the journey to generate embryo-like structures by first trying to see what could be achieved using ESCs alone [16]. They followed a very different strategy to other groups, specifically wanting to avoid the formation of embryoid bodies and so instead of using hundreds of ESCs, they embedded a very much smaller number, comparable to the number of epiblast cells in an individual blastocyst, in Matrigel, a gel of extra-cellular matrix (ECM) components. The idea was that Matrigel would compensate for the missing extra-embryonic cells that normally make the ECM that coats the epiblast, enabling the smaller numbers of ESCs to behave as they would in the implanting embryo. Over a period of three days, they could recapitulate the development of the epiblast at implantation and show the polarization of the ESCs into a three-dimensional rosette-like structure that would subsequently undertake lumenogenesis (figure 2) [16]. This simple model identified the requirement for ECM to stimulate this morphogenetic programme and showed how it was coordinated with cell identity and embryo size—the small numbers of initiating ESCs were crucial to make a polarized rosette of cells. When larger numbers of seeding ESCs are used, pro-ammonitic cavity formation utilizes an alternative mechanism that includes programmed cell death [17]. Subsequently, Shahbazi *et al.* [18] from the Zernicka-Goetz group showed that if ESCs were prevented from leaving the naive state of pluripotency, the cells could polarize and make a rosette, but they could not undertake lumenogenesis to form the pro-amniotic cavity. Cavity formation required exit from the naive state of pluripotency and expression of Otx2, which together with Oct4, could drive the expression of Podocalyxin, the secretion of which initiates lumenogenesis [18].

## 4. The posterior of the embryo organizes independently of the anterior

This first model developed by Bedzhov & Zernicka-Goetz [16] used only ESCs and so was missing signals to induce symmetry breaking, which in natural development occurs in response to the extra-embryonic tissues. When the blastocyst implants, its polar part, where the inner cell mass is located, transforms from a ball of cells into a cylinder (figure 1viii). This so-called *egg cylinder* comprises two abutting compartments, the epiblast and the TE-derived extraembryonic ectoderm (ExE), which are both enveloped by VE derived from the PE of the blastocyst. The ExE is generated from only one part of the trophectoderm, the polar trophectoderm, which overlays the epiblast of the blastocyst. The groups of Hiiragi, Hadjantonakis and Soriano showed that FGF4 expressed by the ICM signals through FGFR1 and FGFR2 to promote and maintain PE identity [19–21] and a role for FGF signalling in TE development had been suggested by the Rossant lab's discovery that FGF4 was necessary to maintain the multipotent character of trophoblast stem cells lines [14]. Christodoulou and colleagues in Zernicka-Goetz group showed that upon implantation, the epiblast provides an FGF signal to its overlying polar-trophectoderm to form the ExE [22,23]. The folding of this part of the TE at this stage also induces spreading of the VE so that it envelops whole of the egg cylinder. The VE at the distal-most tip of this cylinder migrates anteriorly and was known from studies in Rosa Beddington's lab to behave as a signalling centre, the anterior VE (AVE) [24] (reviewed in [25]). Cells of the AVE express inhibitors of signalling pathways, leading to the formation of a morphogen gradient of Nodal, Wnt and BMP, originating from the ExE. The primitive streak then forms in the posterior part of the egg cylinder to initiate gastrulation, in which cells leave the epiblast and migrate to form a layer of mesodermal cells between the epiblast and VE (figure 1ix). The route of VE migration and the positioning of the primitive streak are influenced by asymmetric perforations in the basal membrane discovered by Kyprianou and colleagues in the Zernicka-Goetz group [26]; the AVE migrates along the non-perforated part whereas the perforated part appears to facilitate the ingression of cells from the primitive streak during gastrulation. It was therefore clear from the work of many groups that the extra-embryonic tissues had essential roles in signalling morphogenesis of the pluripotent epiblast upon embryo implantation. The next step was to try and mimic these events in the formation of the egg cylinder. To this end, a strategy was developed to test the consequences of stem cells representing each of the extraembryonic tissues, so determining the role of each type of stem cell.

**Figure 3.**
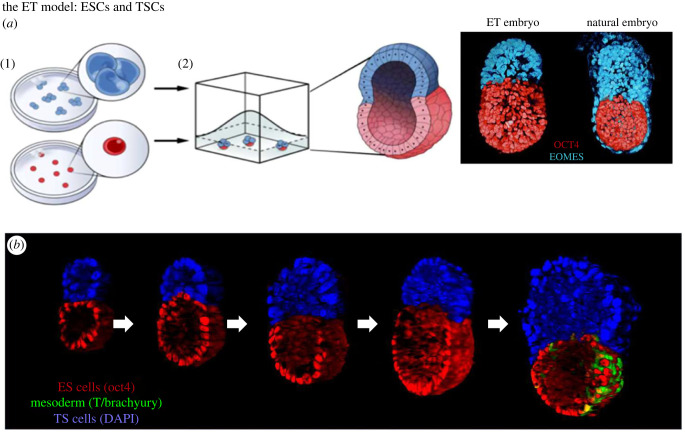
Generation of ET model embryos. (*a*) ESCs and TSCs plated into Matrigel are able to self-assemble into structures resembling the natural embryo at the egg cylinder stage bit noticeably missing the PE-derived, visceral endoderm. (*b*) As the ET cylinders grow and develop, their gene expression patterns indicate formation of mesoderm progenitors and primordial germ cells. The onset of expression of the mesoderm marker, Brachyury, is shown in green as it would in anticipation of gastrulation, which does not take place. The anterior is not specified this system. From [27].

Harrison and her co-workers began this by seeding ESCs and TSCs once again into Matrigel (figure 3). They found that the two cell types sorted from each other and organized themselves into a bicompartmental structure that strongly resembled the epiblast and ExE compartments of the egg cylinder [27]. The resulting ET structures did not have any PE-derived tissue and so would not have been able to develop an anterior signalling centre. Despite this, they strongly resembled embryos at the egg cylinder stage, offering the opportunity to determine the extent to which development could take place if only these two tissues were present. Strikingly, the structures were able to break symmetry and induce mesoderm patterning in the absence of an AVE. By constructing embryos from ESCs that expressed GFP under the control of the mesoderm marker, Brachyury, the group could monitor this symmetry-breaking event and show that cells expressing GFP had gene expression patterns that were extremely similar to the posterior cells of 7-day old gastrulating embryos. By contrast, the cells from the opposite end of the polarity axis were not well specified as anterior epiblast as judged by their gene expression patterns.

This finding has resonance to previous reports of the effects of removing the hypoblast, the visceral endoderm of chick embryos [28] and mouse where in the absence of Cerberus + Lefty the AVE does not form [29]. In both cases, this leads to the development of multiple primitive streaks. Thus, primitive streak formation can be independent of the AVE. A body of work also points to the importance of extra-embryonic tissue in regulating the formation and positioning of the streak in the chick embryo [30].

## 5. Order out of chaos

**Figure 4.**
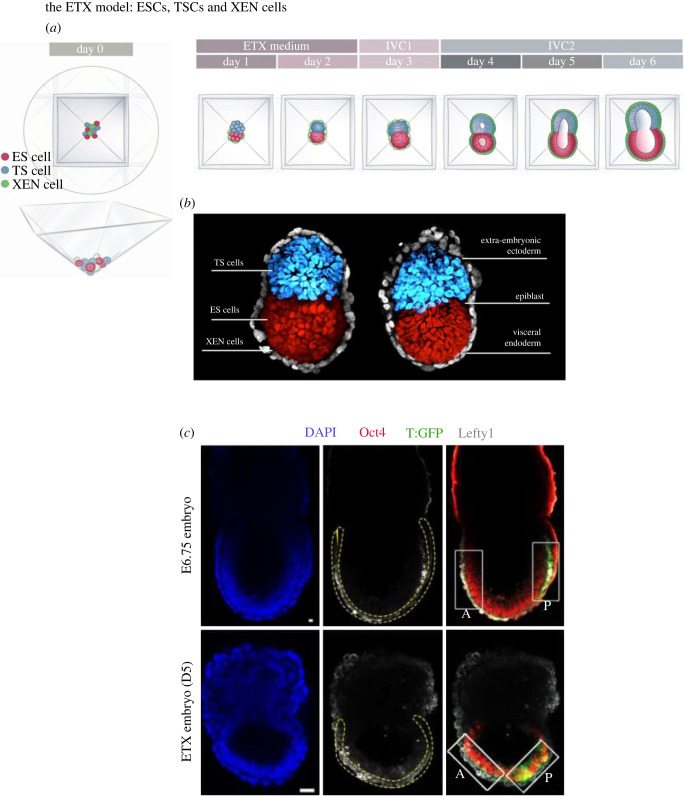
The ETX model. (*a*) ESCs, TSCs and XEN cells are plated onto pyramidal Aggrewells, whereupon they randomly associate with between 10–15% self-assembling into structures resembling post implantation egg cylinders. ETX culture medium is described elsewhere ; IVC media were developed for culture of mouse and human blastocysts through the implantation stages are also described elsewhere [35,36]. (*b*( Side-by-side views of ETX model structures (left) and natural egg cylinders (right). ESCs/epiblast, red; TSCs/TE-derived extra-embryonic ectoderm (blue); XEN cells/visceral endoderm (grey). (*c*) Natural embryos initiate the movement of the Anterior Visceral Endoderm (AVE; Lefty1, grey) and gastrulation, indicated by Brachyury (T-GFP, green) expression in mesoderm at E6.75. By contrast, although progression of the primitive streak (T-GFP, green) is advanced, the AVE (Lefty1, grey) does not migrate fully in ETX model embryos and gastrulation is incomplete. From [31].

**Figure 5.**
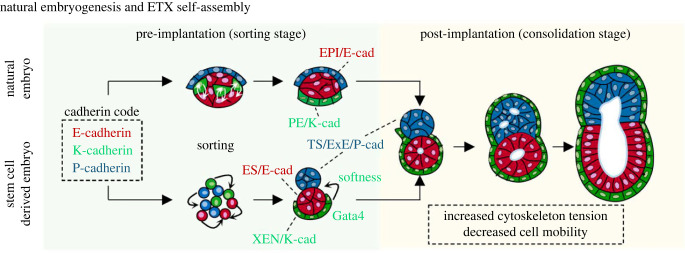
Principles of self-assembly of the ETX model. The three stem cell types each have a specific code of cadherin isoforms on their surfaces. In natural development this enables first, the sorting of epiblast from primitive endoderm in blastocyst formation and second, in establishing a distinct compartment of TE-derived extra-embryonic ectoderm in the egg cylinder. The three cultured stem cell types utilize this cadherin code to self-assemble into compartmentalized structures resembling the egg cylinder stage, thus bypassing the morphological arrangement of the blastocyst. In addition, XEN cells have low cortical tension, which helps them envelop the ESC- and TE-derived compartments. After 24 h, the assembled structure becomes fixed by an increase in cortical tension in all cell types, thereby decreasing cell mobility. A detailed discussion together with *in silico* modelling is given elsewhere [32]. Drawings by Andy Cox and Min Bao.

The above experiments raised the question of whether an anterior signalling centre (resembling the AVE) would develop in the distal most part of the egg cylinder if VE were to be added into the model. To do this, it was necessary to add a third type of stem cell; extraembryonic endoderm or XEN cells to provide the missing PE-derived, tissue. Because the VE lays down the ECM of the basal membrane, Matrigel was no longer required as a part of the culture platform and so a new strategy was needed to bring cells together. To address this, Sozen and colleagues in the Zernicka-Goetz lab plated mixtures of ESC, TSC and XEN cells in a dish with multiple tiny pyramidal wells to provide a geometrical constraint that would facilitate cell interactions. The resulting model, ETX-embryoids, were amazing; placed side-by-side embryos at the egg cylinder stage, they were indistinguishable (figures 4 and 5) [31].

The frequency by which such structures assembled was from one perspective quite low; only around 12–15% of structures assembled correctly into structures indistinguishable from embryos. From another viewpoint this is quite remarkable. Three different cell types had been added to a well and a substantial portion of them could, through random collisions, self-assemble into the correct multicellular structures. Understanding of how this could be achieved has been recently described by Min Bao *et al.* [32]. Two factors were found to be of major importance in the self-assembly of these ETX structures: cell adhesion and cortical tension. Each stem cell type expresses a distinct set of cadherins—ESCs and epiblast cells express E-cadherin, TSCs and ExE cells express both E-cadherin and P-cadherin, and XEN cells express K-cadherin. Homophilic interactions lead like-cells to cluster together and the heterophilic interactions establish a hierarchy of preferred partnerships. There are clearly many possible ways in which the cells can self-assemble into compartmentalized structures—for example a cluster of ESCs could interact with either one or several clusters of TSCs and vice versa. However, notwithstanding the random nature of this self-assembly process, there is another factor influencing the success rate. It turned out that the cultured stem cells do not have unform levels of the cadherin isoforms on their surface. Some ESCs, for example, had high levels of E-cadherin, others had low levels. Similar findings were made for the cadherin isoforms expressed by the other stem cell types. This heterogeneity of cadherin expression levels in the cultured stem cells contributed to the formation of imperfect structures and when the cadherin code in ESCs and TSCs was controlled by uniformly expressing the different cadherins, the frequency of correct cell sorting and self-organization was increased by some 3-fold.

Analysis of the differential expression of E-, K- and P-cadherins revealed another important property of their cadherin codes; the codes indicated the different stem cell types were at a slightly different stage of their development. The XEN-cell cadherin code resembled the code of pre-implantation PE whereas the TSC code resembled more that of the newly formed ExE just after implantation. Thus, the assembly of XEN-cells into a layer below ESCs in the ETX-embryoid seems to recapitulate the corresponding sorting events that separate PE from epiblast in the pre-implantation embryo. On the other hand, sorting of TSCs into a layer above ESCs in the ETX-embryoid resembles the clustering of ExE above the epiblast, which takes place in the natural embryo after implantation.

It is not only adhesion, but also cortical tension that influences the self-assembly process [32]. A decrease in the cortical tension of XEN cells is of particular importance early in the assembly process to achieve the externalization of the XEN cell layer. This loss of cortical tension in XEN cells can be driven by expression of Gata4, a master regulator of PE identity [33]. XEN cells thus become much softer than the other two cell types, allowing the XEN cells to envelop the ESC- and TSC-derived compartments. In fact, the softening of XEN cells contrasts with the increase in cortical tension of the ESCs and TSCs. Jake Cornwall-Scoones was able to model these events *in silico* and show the importance of the increase in cortical tension at the end of the sorting process [32]. This increased tension effectively locks aggregates of the different cell types into place, whether they are correctly sorted or not—if cells don't sort in time, this increased tension leads to the cells becoming ‘jammed’ in the wrong compartments.

This study gives insight into how the three cell types can assemble into the correct structure more efficiently and one can imagine future work to direct the correct placement of cells to facilitate their correct spatial alignments in the assembly process. In practice mis-sorting is not a problem as the plating of the three stem cell types onto an Aggrewell plate generates literally hundreds of correctly assembled egg cylinders. These can easily be identified and picked and once they are correctly assembled at this stage then greater than 90% will proceed to recapitulate development.

## 6. Perfecting gastrulation

**Figure 6.**
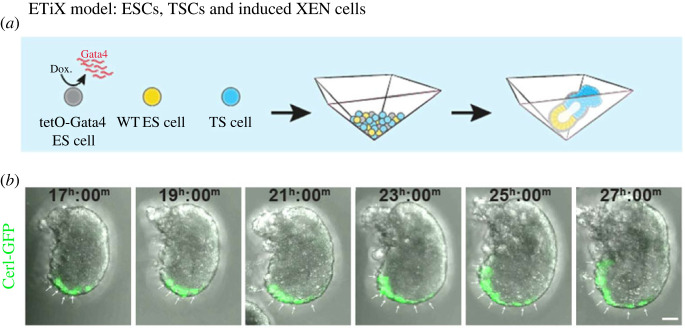
The ETiX model. (*a*) To overcome the limited participation of the synthetic visceral endoderm in both AVE migration and gastrulation, XEN-like cells were generated at an earlier stage of their development by inducing a doxycycline-driven pulse of Gata4 expression in ESCs. This line was substituted for XEN cells in the assembly of ETiX structures in Aggrewells. (*b*) Series of time lapse images showing full anterior migration of the AVE (marked by Cerl-GFP) in the ETiX system. In addition, the gastrulation movements are completed in these structures as described elsewhere [34].

The ETX system was greatly encouraging. The model structures exhibited the two major attributes of anterior-posterior axis formation: an anterior signalling centre could form and the structures could initiate the epithelial-mesenchymal transition (EMT) cell movements of gastrulation, whereby epiblast cells migrate to form a separate layer of mesodermal cells sandwiched between the epiblast and the VE. However, the AVE did not migrate and gastrulation movements were not fully executed. The solution was found by Amadei and colleagues, who established a new principle for providing extra-embryonic cells, in this case XEN cells, with ESCs in which the transient expression of the PE-determining transcription factor, Gata4, could be induced (figure 6*a*) [34]. This generated stem cells better resembling younger visceral endoderm. When these Gata4-inducible ESCs (iXEN cells) were combined with TSCs and wild-type ESCs, they collectively self-organized into ETiX-embryoids with the correct compartments that could develop a robust anterior signalling centre that migrated towards the future anterior, while on the opposite site, a posterior domain formed that undertook complete, successful gastrulation (figure 6*b*).

## 7. One more day!

Could the gastrulating embryoids formed using induced XEN cells develop further? With this question in mind, the Zernicka-Goetz lab revisited previous studies for post-implantation embryo culture. The laboratory had previously developed so-called IVC media that enabled both natural mouse and human embryos to develop from blastocysts through to gastrulation in the culture dish [35,36]. Other media were, however, required to take development further. Fortunately, there was a rich history of such studies. Techniques had been developed by Dennis New in the 1970s to culture rat embryos *ex utero* to the head-fold stages (0–5 somites) after nearly 4 days in culture [37,38]. To attain similar ends with *ex utero* mouse embryos, Patrick Tam had tested a variety of media in the early 1980s and concluded that Dulbecco's modified Eagle's Medium (DMEM) with additional glucose and glutamine being critical together with correctly prepared mouse or rat serum present at greater than 50% to achieve development to the somite stage [39]. In the following years, Tam further refined the medium and showed that a mixture of 25% DMEM; 50% rat serum and 25% human cord serum (DRH medium) [40,41] allowed embryos to develop multiple somites when transferred into the rotating bottle system developed by Dennis New (available through BTC Engineering, Cambridge, UK). Kazuhiro Eto's group also developed a similar rotating bottle incubator (available through Ikemoto Co., Japan) and described their ability to grow rat embryos from E9.5 (i.e. corresponding to E7.5 in mice) for 96 h up to the stage with 40-somites when the hindlimbs appear. Embryos were cultured in 100% rat serum from initially very low concentrations of oxygen increasing up to 95% [42,43]. More recently, Jacob Hanna's laboratory have described how they combined existing techniques and transferred gastrulating embryos from a modified and renamed Zernicka-Goetz IVC medium into DRH medium (now buffered with Hepes rather than bicarbonate, lacking phenol red as pH indicator and renamed EUCM) for culturing mouse embryos into the BTC version of New's rotating bottle incubator but adding a device to supply specific mixtures of pressurized gases. The combination of these culture techniques allowed continued development from before gastrulation (E5.5) until the hindlimb formation stage (E11) [44]. It remains of interest to test Eto's incubator under comparable conditions. Jacob Hanna kindly provided the Zernicka-Goetz group with such a device to use in the identical rotating bottle culture incubators, which had been in the Cambridge Physiological Laboratories for the culture of mouse and rat embryos since the 1980s. Both this device modified to provide only the means of delivering pressurised gas and one built in house from commercially available components [45] to facilitate gas flow into the incubator, gave remarkable ETiX embryoid development over the next 24 h as we will discuss below.

## 8. Development of progenitors for all brain regions in the embryoid models

**Figure 7.**
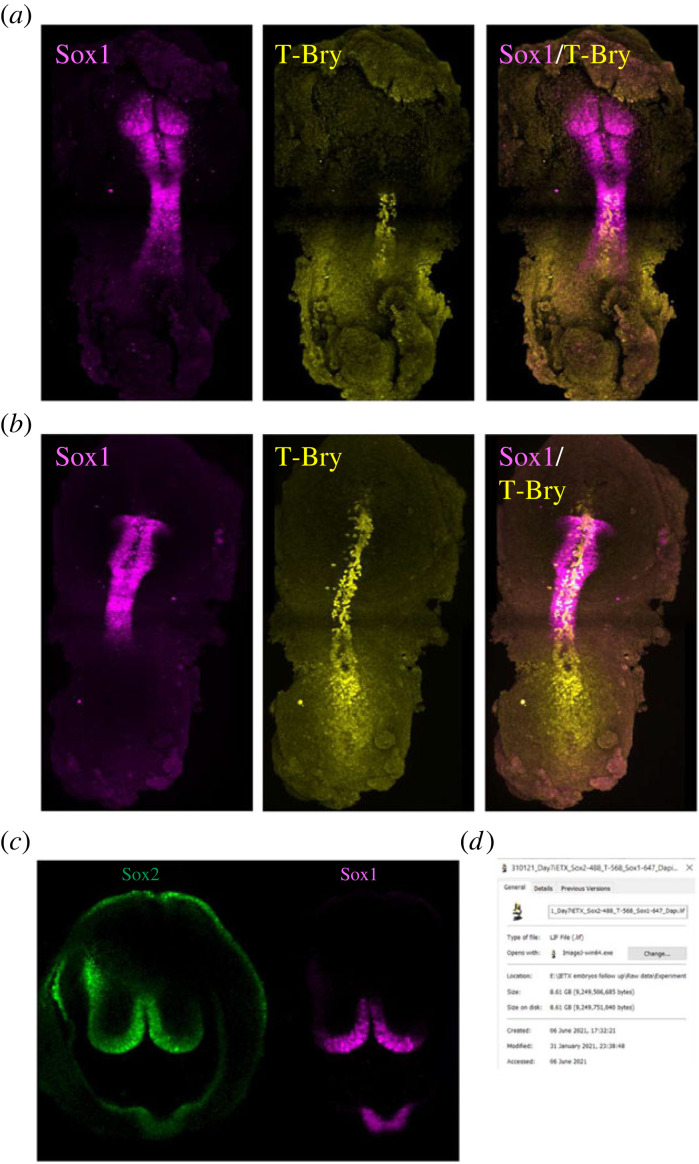
ETiX model embryos initiate brain development in stationary culture. (*a*) Dorsal view of ETiX structures at day 7 of development in static culture showing expression of neuroectodermal marker Sox1in head folds and in neural tube tissue extending extending two thirds the length of the stem cell-derived embryo. Brachyury (T-Bry) -positive posterior exhibits a tail bud-like morphology. (*b*) Ventral view showing Sox1 and T-Bry staining at day 7. Brachyury (T-Bry) positive notochord extends beneath the Sox1 positive neural tube tissue. (*c*) Dorsal view of Sox1 and Sox2 positive cells in headfolds of day 7 ETiX embryo model grown in static culture. (*d*) Date stamp of experiment by GA.

**Figure 8.**
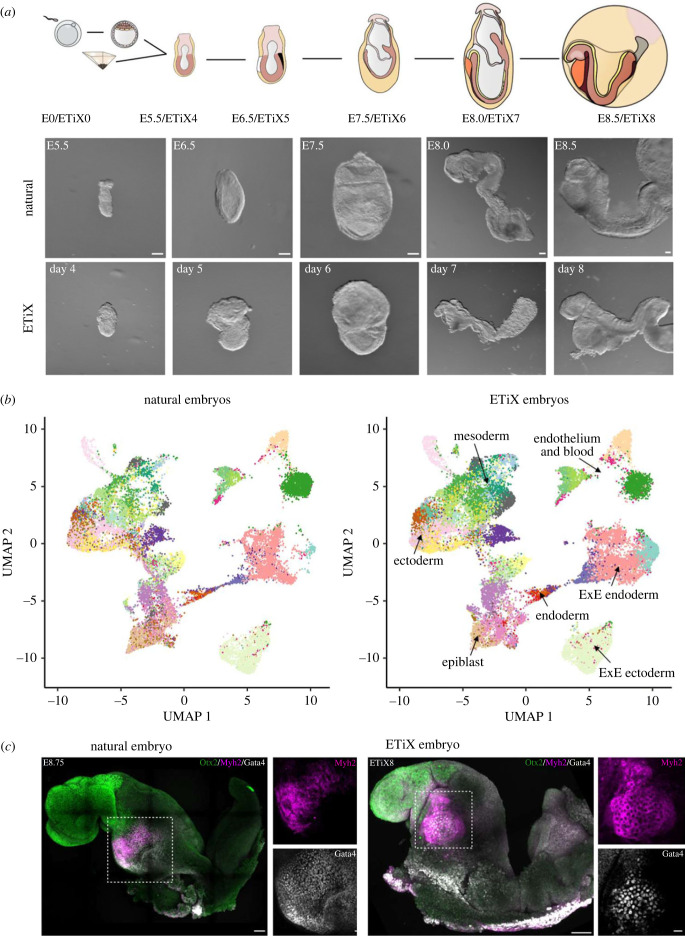
ETiX model embryos develop to neurulation. (*a*) Schematic showing how the egg cylinder structure can be formed during natural development or from three cultured stem cell types, as shown in figure 6. Brightfield micrographs showing development of the resulting structures are shown below until day 8 of culture, which is equivalent to E8.5 of natural development. Note that both the embryo and its model develop within a yolk sac, derived from the extra-embryonic endoderm. The yolk sac is shown in the schematic but has been dissected away from the actual embryos/models. (*b*) Principal component analysis of single cell RNA seq data from natural embryos and from the ETiX model illustrating the accurate representation of multiple cell types in the natural and synthetic structures. The multiple different cell types have been given a colour code, the key for which is given in electronic supplementary material, figure S1. (*c*) Natural and stem-cell-derived model embryos at comparable stages. The model structures have developed progenitors for the fore-, mid- and hind-regions of the brain revealed by Otx2 expression (green). At this stage they have around 4 or 5 pairs of somites (not shown). Myh2 staining (Magenta) is predominantly in the heart. From [46].

It was May 2020 at the beginning of the Covid-19 lockdown when the Zernicka-Goetz group submitted their work showing gastrulation in ETiX embryoids incorporating extraembryonic endoderm from ESCs [34]. They were soon asking whether the stem cell-derived ETiX embryo models would develop further, and to the group's excitement found by January 2021 that by day 7 of culture in stationary conditions, the ETiX-embryoid model could develop an anterior-posterior axis with neural folds that extended into a neural tube and thence to a tail bud joined with allantois tissue connected to the developing chorion and the whole being contained a fluid-filled yolk sac. The structure looked just like the early headfold-stage of an E8.0 natural embryo (figure 7). The next eight months were a roller coaster ride of exciting findings submitted to Nature in November 2021 [46]. The five referees were rightfully demanding to know more about spatio-temporal aspects of gene expression in the multitude of tissues. The further eight months of study provided a detailed compendium of the transcriptional regulation operating in the ETiX embryoids to recapitulate the great majority of cellular events in the development of the natural embryo (figure 8).

The most striking feature of the neurulating ETiX-embryoids, setting them apart from gastruloid systems was their ability to recapitulate development of all brain regions [46]. At day 7, neuroectodermal markers, Sox1 and Sox2, were expressed in neuroepithelial cells, along the entire anteroposterior axis of the neurulating embryoids and a Brachyury-positive notochord ran below the neural tube in a pattern mirroring the natural E8.0 embryo. At day 8, following transfer of the embryoids into Dennis New's rotating bottles with a gas delivery device, the transcription factor Otx2, which contributes to patterning of the midbrain and forebrain was expressed in the anterior-most third of the head-folds of the ETiX-embryoids just as occurs in natural mouse embryos at E8.5. Single-cell RNA seq confirmed that the embryoids expressed appropriate markers of the neural tube with correct spatio-temporal specification and that the neural cell types appeared in line with the major burst of neural induction known to occur in natural development at E8.0. Development of the anterior regions of the central nervous system had not proved possible in the gastruloid systems and although a study to aggregate naive ESCs with an ectopic morphogen signalling centre had given some development of brain progenitors, this had only been of the posterior mid-brain [47]. The success of the ETiX-embryoid in generating progenitors of the fore- and mid-brain regions emphasizes the importance of extraembryonic endoderm in specifying the anterior and protecting it from the posteriorizing influence of the primitive streak in the gastrulating embryo.

In revising the manuscript, the group knocked out *Pax6* in the ESC line used to build the embryoids to test the consequences for neural tube patterning in embryoids. In line with the known mouse knock-out phenotype [48], there was no change in number of Sox1-positive cells in the neurectoderm but there was an increase in Nkx2.2-positive cells and an enrichment of transcripts associated with increased neurogenesis [46]. This validation of a known genetic defect in natural development is a pointer towards how one can expect synthetic systems to be used in future to help us gain understanding of the developmental process while reducing our reliance upon experimental animals.

## 9. Somites, heart, gut tube and germ cell development

The ETiX-embryoids also displayed the expected gene expression pattern of neuromesodermal precursors, which give rise to derivatives of the neural tube and paraxial mesoderm [46]. As in natural embryo development at E8.0, the paraxial mesoderm of day 7 embryoids gave rise to somites, the paired blocks of cells that form along the anterior-posterior axis of the embryo and which are required for segmental formation of skeletal muscle, blood vessels and skin. By day 8, the embryoids had beating hearts beneath the encephalon region. These structures expressed Myosin heavy chain II (Myh2) and the transcription factor Gata4, which are required for cardiac development, in a similar spatiotemporal profile to the natural embryo. Together, immunostaining and scRNA-seq data showed that neurulating embryoids expressed markers of the developing heart fields and with atrial and ventricular markers just as seen in natural embryos.

Gene expression signatures also revealed the initiation of gut tube development from definitive endoderm and VE gut progenitor cells. Markers for the specific organs of the gut tube were not expressed in line with the developmental stage reached by the embryoids and the known onset of organ-specific identities in the gut tube at a later stage (E8.75)^.^ It will be of considerable future interest to determine whether development of the stem cell-derived embryo models can be extended beyond this stage.

The primordial germ cells were another matter. These repositories of the genome for the next generation are set aside early in embryonic development. Indeed, it was already known from the study by Harrison *et al.* [27] that primordial germ cells could form even in the ET embryo model. Now, it was possible to track PGCs in later timepoints of ETiX-embryoid development where they could be found in proximity to the allantois at days 7 and 8, similar to their positioning in natural embryos at E8.0 [46].

## 10. Importance of the extraembryonic tissues

A really striking feature of the embryoids was that they developed inside membranes that resembled the amnion and yolk sac, whose natural role is to provide nourishment to the embryo until the foetal-maternal circulation becomes established. The presence of these structures reflects the participation of extra-embryonic tissues, specifically the extraembyonic endoderm, in the assembly of embryoids to give rise to both yolk sac and amnion cells. Not only did the ETiX embryoids develop a yolk sac but their extraembryonic portion supported haematopoiesis just as in natural development. This was evident from the formation of Runx1-positive blood islands in the mesoderm of the yolk sac and at the base of the allantois of neurulating embryoids at day 8 [46]. In accord with the formation of blood islands, genes associated with endothelial cells of blood vessels were also expressed.

However, the parietal/visceral endoderm-derived lineages were not perfect in the embryoids as some of these cells were at an immature stage in their developmental programme. Such cells began to appear in the day 6 embryoids, but the vast majority of these potentially aberrant cells appeared at day 8. The lineages derived from trophectoderm were also not perfect. During natural development, the proximal portion of the ExE differentiates into the ectoplacental cone (ECP), precursor of trophoblast giant cells and spongiotrophoblasts and although markers of these cells could be detected, the ECP lineage was not fully developed even though development of the chorion lineages had largely taken place.

A further indication of the importance of the correct specification of trophectoderm-derived tissue came from graduate student Kasey Lau in the Zernicka-Goetz lab, working in collaboration with Yonatan Stelzer's lab at the Weizmann Institute. Kasey had made embryoids from three stem cell components in which not only extraembryonic endoderm was generated from ESCs, but also TSC-like cells. In the latter case, ESCs were induced to develop into TE-like cells by expression of the TE-determining transcription factor, Cdx2. Strikingly these EiTiX embryoids also developed into structures at the head-fold stage with neural tubes, somites and beating hearts [49]. Unknown to Kasey and the team, Jacob Hanna's laboratory in the Weizmann Institute had also begun to independently generate and had followed a similar route to Kasey. Gratifying, the Hanna laboratory had an almost identical set of findings so validating the experimental system in a different laboratory [50].

It seemed that the embryoids in which both extraembryonic lineages were derived from ESCs developed to the neurulation stages at a lower efficiency. Moreover, the Hanna group also found that their equivalent of ETiX embryos developed better than EiTiX embryos [50]. Clues as to why this might be so came from the RNAseq analysis of Kasey Lau's EiTiX embryoids by Hernan Rubinstein in the Stelzer laboratory. The vast majority of i*Cdx2* ESCs (97.26%) gave rise to chorion lineage and the small number that did not, appeared not to have been induced to differentiate [49]. The EPC and TGC subtypes could not be detected in either Day 6 or Day 8 EiTiX-embryoids indicating that i*Cdx2* ESCs have restricted differentiation potential. In natural embryos, *Sox2*, *Cdx2* and *Eomes* are downregulated in the distal part of the ExE, where the EPC and TGC subtypes are found [51] but in Day 5 EiTiX-embryoids, the expression of these genes was retained throughout the ExE compartment. The lack of the EPC and TGC cell types seems to have consequences not only for development of extraembryonic tissues but also for embryo development as some differentiated embryonic cell types were missing in both Day 6 and Day 8 EiTiX-embryoids and the synchronicity between lineages was impaired [49].

## 11. What next?

The lack of EPC and TGC cell types in EiTiX embryoids is a shortcoming of this particular system. It could arise partly because EiTiX-embryoids lack the interactions with the maternal environment that they would have *in utero*; and partly because transcription factor-mediated induction biases *iCdx2* ESCs to differentiate into chorionic cell types. To correctly represent TE-derived cells in development, it might be necessary either to induce expression of genes promoting ExE differentiation or to downregulate TSC-like genes to generate EPC and TGC subtypes in EiTiX embryo. Understanding of the precise roles of such cells in the developmental process needs future engineering.

The extra-embryonic tissues not only need to have correct interactions with the developing epiblast to promote correct development, but they must also develop to form the correct structures that interface with the mother. The current embryoid models built from embryonic and extra-embryonic stem cells take the embryoids to a stage at which, in natural development, the embryo would become reliant upon the developing placenta. One of the next challenges will be to develop ways of substituting for these placental functions to enable continued development. Extrapolating these systems to other mammals, particularly humans, will present a considerable challenge because of the diversity of placental structures. Moreover, the spatial organization of the implanting human embryo differs markedly from the mouse in that it is a disc-like structure, as opposed to a cylinder, and within this disc there is an additional cellular compartment, the amnion, which is derived from the pluripotent epiblast. Thus, we might anticipate distinct differences in the pathways of morphological remodelling and molecular signalling between these tissues throughout the post-implantation developmental stages of mouse and human that will be fascinating to unravel.

## Data Availability

Figure S1 is provided as electronic supplementary material [52].
