# Correction to: ‘Behind the developing brains and beating hearts of stem cell-derived embryo models’ (2023) by Amadei and Glover

**DOI:** 10.1098/rsob.230034

**Published:** 2023-03-08

**Authors:** Gianluca Amadei, David M. Glover

*Open Biology* **13**, 220325. (Published online 11 January 2023.) (https://doi.org/10.1098/rsob.220325)

The conflict of interest disclosures should have read as follows:

Conflict of interest declaration. D.M.G. is a member of the *Open Biology* Editorial Board and is the spouse of Magdalena Zernicka-Goetz. As an author of the above Commentary article, D.M.G. had no involvement in the peer review or editorial decision-making, which was handled by Subject Editor Marek Mlodzik. G.A. declares no conflicts of interest.

This has been corrected on the publisher's website.

